# Supplementary material for: Waning Humoral Response after COVID-19 mRNA Vaccination in Maintenance Dialysis Patients and Recovery after a Complementary Third Dose
Source: Vaccines (Basel). 2022 Mar 11;10(3):433. doi: 10.3390/vaccines10030433 (PMC8950255; doi:10.3390/vaccines10030433)
Supplement: Supplementary file 1 [file vaccines-10-00433-s001.zip › vaccines-1603181-supplementary.pdf]

Figure S1: Comparison of anti-S IgG titer after 3rd dose between IN-D vs. PI-D adjusted to sex, age, BMI, CCI. Multivariable analysis (ANCOVA p-value for the model <0.01). Continuous variables are square root transformed.

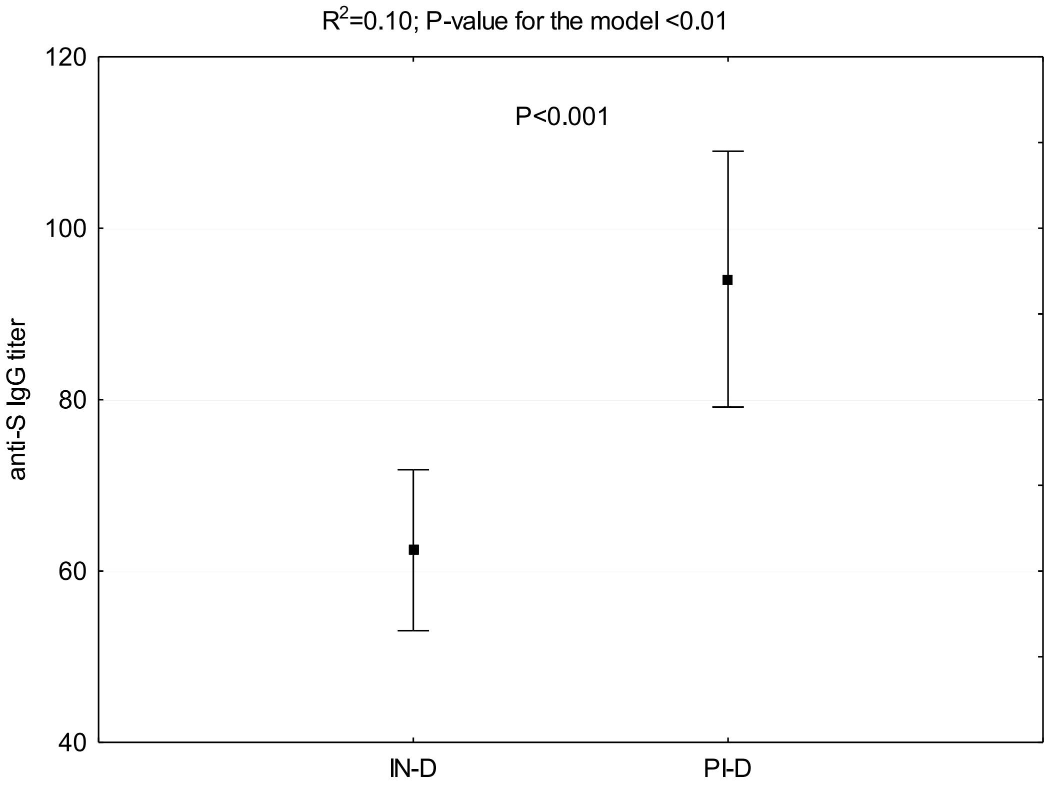

	(ß)	SE (ß)	lower 95%CI	upper 95%CI	p-value
Intercept					0.14
Age	0.06	0.08	-0.10	0.22	0.46
BMI	-0.07	0.11	-0.30	0.15	0.52
CCI<5	-0.05	0.11	-0.28	0.17	0.64
Sex (F)	-0.05	0.08	-0.22	0.11	0.52
Study group (IN-D)	**-0.33**	**0.08**	**-0.49**	**-0.16**	**<0.001**
